# King Edward's Hospital Fund Bill

**Published:** 1907-06-15

**Authors:** 


### KING EDWARD'S HOSPITAL FUND BILL.

Last week we reported the proceedings of the Committee of the House of Commons on Unopposed Bills in regard to this Bill. The Chairman, Mr. Caldwell, and other members objected to the Bill on the grounds that the whole management of the Fund was given to one person, that the same power was given to future presidents as that to be confided in the Prince of Wales, and that the president was irremovable. The committee took no objection to the methods of selecting future presidents but they held it might not always be possible to have a person who would enjoy the same measure of public confidence as the present Prince of Wales. The chairman suggested that the exceptional powers to be given under the Bill might be maintained for the present president but that no such power should be enjoyed by future presidents. All these matters are to be rediscussed and determined after we go to press at the adjourned meeting of the committee on the 13th instant.

We understand, however, that the objections raised are to be met by carefully considered amendments which provide: (1) That when a vacancy occurs in the office of president, the Lord Chancellor, the Prime Minister and the Governor of the Bank of England shall recommend a successor to the Sovereign, that successor being a son, brother, or grandson of the Sovereign. (2) In the event of no such appointment being made, they shall recommend three governors to exercise the powers of the president, and to hold office in commission. (3) The General Council shall, so far as may be practicable, consist of never less than twelve members, each of whom shall be secure in his office for the year of his appointment, and be eligible for re-election. The second amendment provides against the contingency of there being at any time no son, brother or grandson of the Sovereign eligible for the post of president.

Most of the criticism which the Bill has excited has arisen from misapprehension. The constitution as embodied in the Bill is that under which the Fund has worked since its inception. It is not sufficiently realised that the King's Hospital Fund was in fact created by His Majesty when Prince of Wales, with the sanction of Queen Victoria, to commemorate her Diamond Jubilee. It is further to be remembered that much of the success of the Fund has been due to the sustained interest of the King and the Prince of Wales, both of whom have rendered considerable personal service in the work of the Fund, and to them belongs in large measure the credit of raising much of the money which is now invested, say one million pounds. All this money has been given by the public to the Prince of Wales as president of the Fund, and the contributors of the money who constitute its members are entitled to promote a private Act of Parliament to incorporate the Fund, and to provide for its administration upon a plan which has commended itself to their approval. The present strong position of the King's Hospital Fund for London testifies to the approval of the members. We hope that the Bill may be accepted by the House of Commons, and that it may now pass its remaining stages without further objection or difficulty.

